# Deep Learning-Based Imaging Analysis Reveals Radiation-Induced Bystander Effects on Cancer Cell Migration and the Modulation by Cisplatin

**DOI:** 10.3390/ijms26167822

**Published:** 2025-08-13

**Authors:** Ryosuke Seino, Hisanori Fukunaga

**Affiliations:** Department of Biomedical Science and Engineering, Faculty of Health Sciences, Hokkaido University, N12 W5 Kita-ku, Sapporo 060-0812, Japan; seino.ryosuke@hs.hokudai.ac.jp

**Keywords:** cell migration, chemoradiotherapy, cisplatin, non-targeted effect, radiation-induced bystander effect

## Abstract

Regulating tumor invasion and metastasis is pivotal for improving cancer patient prognosis. While cell migration is a key factor in these processes, the non-targeted effects of chemoradiotherapy on cell motility remain poorly understood. In this study, we employed HeLa-FUCCI cells—a cervical cancer-derived HeLa cell line integrated with the Fluorescent Ubiquitination-Based Cell Cycle Indicator (FUCCI) probe, enabling the visualization of cell cycle phases—to investigate the radiation-induced impacts, including non-targeted effects, on cell migration. To create irradiated (In-field) and non-irradiated (out-of-field) regions, half of the culture dish was shielded with a lead block during irradiation. Cells were then exposed to 2 Gy X-rays, with or without cisplatin. Following irradiation, the cells were subjected to time-lapse imaging at 15 min intervals for 24 h, and the acquired data were analyzed using cell segmentation and tracking algorithms, Cellpose 2.0 and TrackMate 7. Without cisplatin, the migration velocity and total distance traveled of Out-of-field cells were significantly reduced compared to controls, suggesting a suppressive bystander signal. In contrast, with cisplatin treatment, these parameters significantly increased in both In-field and Out-of-field cells. This suggests that chemoradiotherapy may inadvertently enhance tumor cell motility outside the target volume, a critical finding with significant implications for therapeutic outcomes.

## 1. Introduction

Unraveling and regulating the mechanisms of the activation of invasiveness and metastasis, one of the hallmarks of cancer [1], is an indispensable process as a cornerstone in the advancement of cancer treatment strategy because metastases account for nearly 90% of cancer-related deaths [2]. Cell migration is thought to be closely involved in tumor invasion and metastasis [3]. While radiation is known to enhance cell migration [4], the impact of non-targeted effects, including the bystander effect during chemoradiotherapy, on this process remains poorly understood. This uncertainty poses a major challenge in the control of tumor invasion and metastasis following chemoradiotherapy.

We previously reported the development of a deep learning-based imaging analysis framework that integrates cell segmentation and tracking algorithms, enabling the elucidation of cell cycle-dependent migration activity following X-ray exposure [5]. We employed HeLa-FUCCI cells, a HeLa cell line derived from cervical cancer, into which the fluorescent probe Fluorescent Ubiquitination-Based Cell Cycle Indicator (FUCCI), which is capable of visualizing the cell cycle, was introduced [6]. We also used the combination of Cellpose 2.0, a deep learning-based algorithm for cell segmentation [7,8], and TrackMate 7, an automated tracking software application that enables the tracking of target cells from two-dimensional (2D) microscopic bright-field images [9,10,11]. Notably, the migration velocity of G1-phase-synchronized cells at the time of irradiation exhibited a dose-dependent decrease, whereas G2-phase-synchronized cells showed a tendency for increased velocity. Further, cells arrested at the G2 phase by the treatment of cisplatin, a Pt-based anticancer agent [12], exhibited increased cell migration after irradiation. These findings suggest that anticancer agents capable of arresting cancer cells at specific phases of the cell cycle may inadvertently promote tumor invasion and metastasis following chemoradiotherapy. However, the non-targeted effect following chemoradiotherapy on cancer cell migration remains unelucidated. Radiation-induced non-targeted effects, particularly the bystander effect, have long been suggested to contribute to the efficacy of cancer treatment [13].

**Figure 1:**
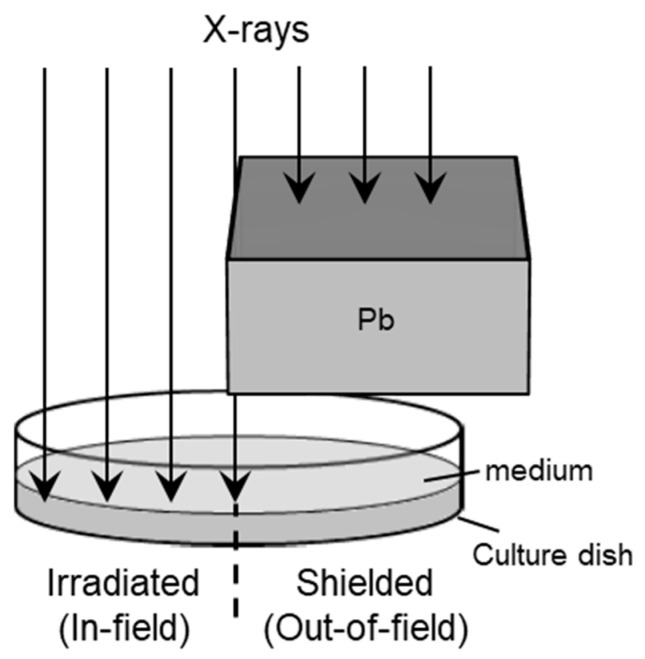
Schematic representation of the half irradiation field. Cells were irradiated at 2 Gy in a single 60 mm culture dish.

To approach the unresolved issue, in this study, HeLa-FUCCI cells were seeded in culture dishes in which half of the area was shielded during irradiation, thereby creating an irradiated area (In-field) and a non-irradiated area (Out-of-field) (Figure 1). In addition, the cells were exposed to 2 Gy of X-rays with or without cisplatin treatment. Following irradiation, time-lapse imaging was performed at 15 min intervals for 24 h. The acquired images were subjected to deep learning-based imaging analysis using cell segmentation and tracking algorithms to evaluate cell migration velocity and direction. This approach was anticipated to be capable of detecting and analyzing subtle changes that would otherwise be imperceptible to the human naked eye.

## 2. Results

### 2.1. Cell Migration Activity in Spatially Fractionated Radiation Fields

**Figure 2:**
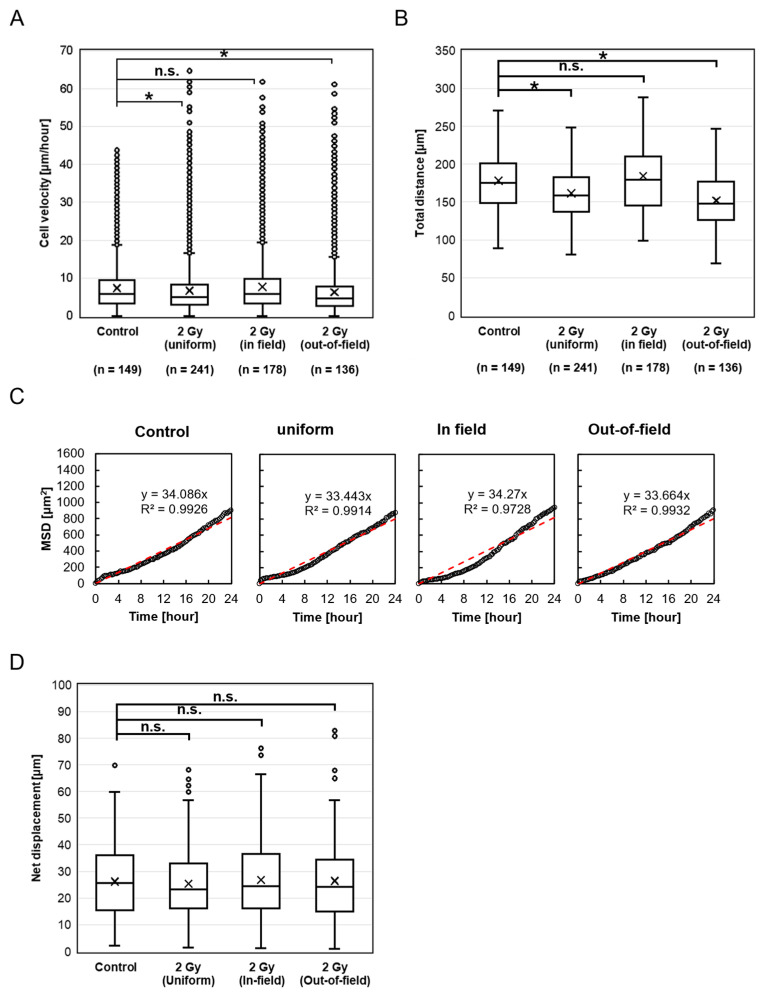
HeLa-FUCCI cell migration after exposure to X-rays. (**A**) Box-and-whisker diagrams showing the distribution of migration velocity in the Control, Uniform, In-field, and Out-of-field cells. * denotes *p* < 0.05; n.s., not significant. (**B**) Box-and-whisker diagrams showing the total distance traveled by individual cells over the observation period. * denotes *p* < 0.05; n.s., not significant. (**C**) Correlations between the time after irradiation and the mean square displacement (MSD). All MSDs can be described as linear for at least 24 h after irradiation. Red dashed lines indicate the results of linear fitting. (**D**) Net displacement, defined as the straight-line distance between the starting and ending positions of each cell. n.s., not significant.

We assessed the migration velocity of HeLa-FUCCI cells following exposure to 2 Gy X-rays across four groups: Control, Uniform, In-field, and Out-of-field (Figure 2A). The migration velocities, expressed as median (interquartile range), were as follows: Control, 5.73 μm/h (3.31–9.49); Uniform, 4.99 μm/h (2.90–8.36); In-field, 5.86 μm/h (3.35–9.78); and Out-of-field, 4.66 μm/h (2.68–7.84). Notably, migration velocities in the Uniform (*p* < 0.001) and Out-of-field (*p* < 0.001) groups were significantly reduced compared to the Control, whereas the In-field group showed no significant difference (*p* = 0.117).

A similar trend was observed for the total distance traveled, which reflects the cumulative path length of cell movement. Total distance was significantly lower in the Uniform (*p* < 0.001) and Out-of-field (*p* < 0.001) groups compared to the Control, with no significant change in the In-field group (*p* = 0.541) (Figure 2B). To further characterize migration dynamics, we analyzed the mean squared displacement (MSD) over time. In all groups, MSD increased approximately linearly with time (Figure 2C), indicating that the cells exhibited random walk migration patterns. We also evaluated the net displacement, defined as the straight-line distance between the starting and ending positions of each cell. In addition, we evaluated the net displacement, defined as the straight-line distance between the starting and ending positions of each cell. No significant differences in net displacement were observed among the groups: Uniform (*p* = 1.000), In-field (*p* = 1.000), and Out-of-field (*p* = 1.000), compared to the Control (Figure 2D).

### 2.2. Cell Migration Activity with Cisplatin Treatment

**Figure 3:**
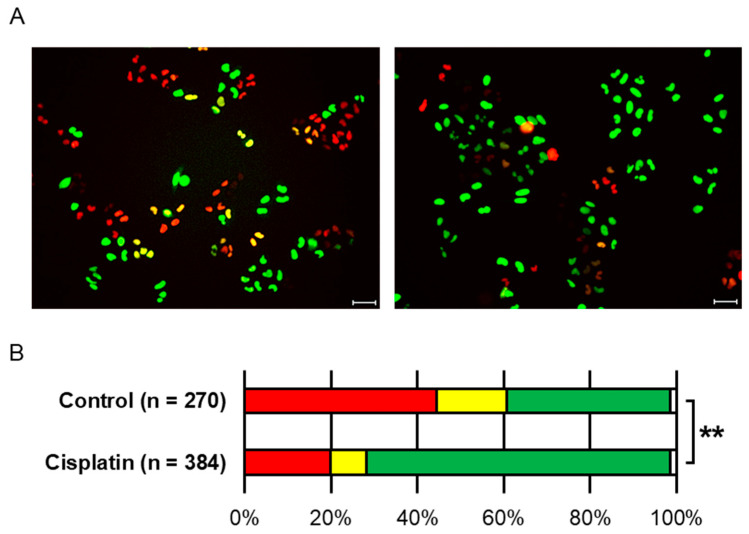
Fluorescent color distributions in HeLa-FUCCI cells. (**A**) Representative fluorescent microscopy images of HeLa-FUCCI cells without cisplatin treatment (**left**) and after treatment with 5 µM cisplatin for 1 h followed by a 14 h incubation (**right**). The scale bar indicates 50 μM. In FUCCI imaging, G1-phase nuclei appear red, early S-phase nuclei appear yellow, and late S–G2/M-phase nuclei appear green. (**B**) Summary graph of fluorescent color distribution in the control and cisplatin-treated groups. The color of the bars indicates the color of the cells. ** denotes *p* < 0.001 (chi-square test).

Figure 3A displays fluorescent microscopy images of HeLa-FUCCI cells without cisplatin treatment (left) and those treated with 5 µM cisplatin for 1 h followed by a 14 h incubation (right). Under a fluorescence microscope, nuclei typically appear red in G1 phase, yellow in early S phase, and green in S to G2 phases [6]. Figure 3B summarizes the distribution of fluorescent colors indicating cell cycle phases. A significant difference in cell cycle distribution was observed between the cisplatin-treated group and the untreated group (** denotes *p* < 0.001, chi-square test). In the cisplatin-treated group, green-fluorescent cells accounted for approximately 70.3% of the total cell population, indicating that a majority of cells were arrested in the G2 phase.

**Figure 4:**
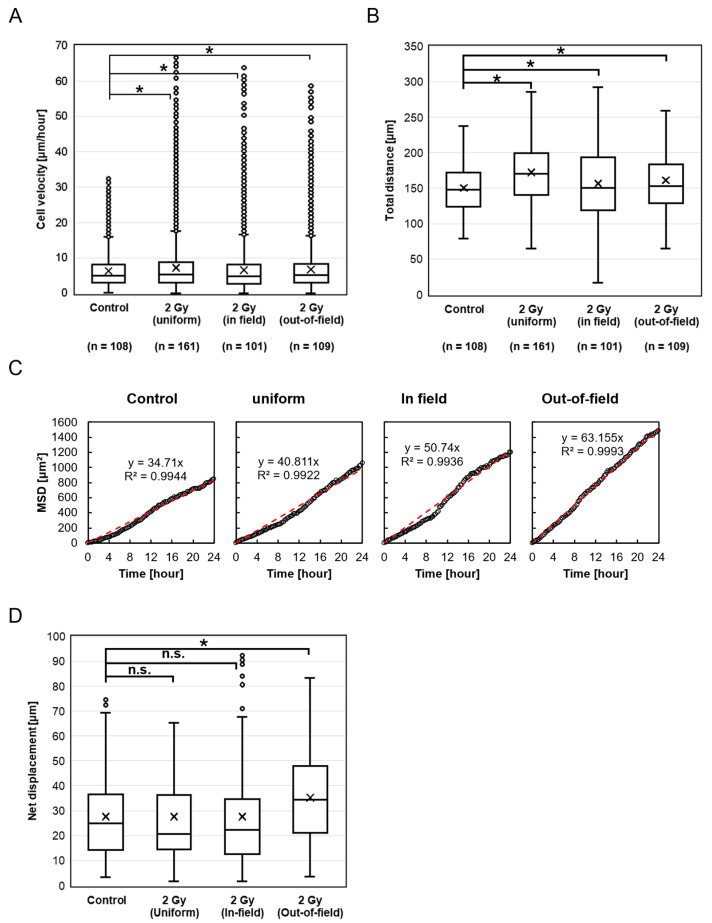
HeLa-FUCCI cell migration after exposure to X-rays and cisplatin. (**A**) Box-and-whisker diagrams showing the distribution of migration velocity in the Control, Uniform, In-field, and Out-of-field cells. * denotes *p* < 0.05. (**B**) Box-and-whisker diagrams showing the total distance traveled by individual cells over the observation period. * denotes *p* < 0.05. (**C**) Correlations between the time after irradiation and the mean square displacement (MSD). All MSDs can be described as linear for at least 24 h after irradiation. Red dashed lines indicate the results of linear fitting. (**D**) Net displacement, defined as the straight-line distance between the starting and ending positions of each cell. * denotes *p* < 0.05; n.s., not significant.

Figure 4A shows the migration velocity of HeLa-FUCCI cells following treatment with cisplatin and exposure to 2 Gy X-rays in the Control, Uniform, In-field, and Out-of-field groups. The migration velocities were as follows: Control, 4.83 μm/h (2.66–8.11); Uniform, 5.28 μm/h (3.05–8.86); In-field, 5.03 μm/h (2.92–8.21); and Out-of-field, 5.17 μm/h (3.04–8.32). Interestingly, all irradiated groups—Uniform (*p* < 0.001), In-field (*p* = 0.002), and Out-of-field (*p* = 0.002)—exhibited a significant increase in migration velocity compared to the Control. A similar trend was observed for the total distance traveled, which was significantly greater in the Uniform (*p* < 0.001), In-field (*p* < 0.001), and Out-of-field (*p* < 0.001) groups than in the Control (Figure 4B). As shown in Figure 4C, cell migration in all groups followed a random walk pattern. Also, there were no significant differences in net displacement between the Control and either the Uniform (*p* = 1.0) or In-field (*p* = 1.0) groups. However, the Out-of-field group showed a significantly increased net displacement compared to the Control (*p* < 0.001) (Figure 4D).

## 3. Discussion

**Figure 5:**
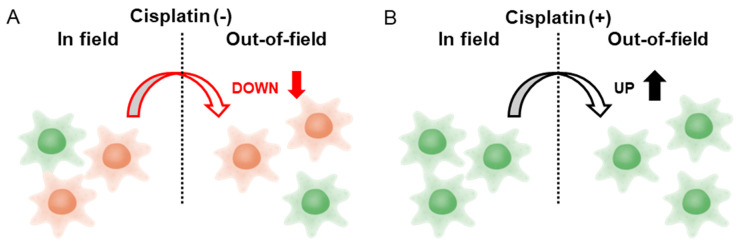
Radiation-induced bystander effects on cancer cell migration and the modification by cisplatin. Red cells indicate G1 phase, and green cells indicate G2 phase. (**A**) Without cisplatin, a signal that decreases the velocity of cell migration from the In-field to Out-of-field cells appears to be transmitted, while a signal that increases the velocity from the Out-of-field to In-field cells is transmitted. (**B**) Cisplatin treatment seems to signal an increase in the velocity of cell migration from the In-field to Out-of-field cells, while signaling from the Out-of-field to In-field cells remains unclear.

This study demonstrated a distinct difference in HeLa-FUCCI cell migration velocity after X-ray irradiation with or without cisplatin treatment. In the absence of cisplatin, both the Uniform and Out-of-field cells exhibited a significant decrease in migration velocity after irradiation, compared to the Control, whereas no such decrease was observed in the In-field group. Similarly, both the total distance traveled by Uniform and Out-of-field cells significantly decreased after irradiation compared to the Control, whereas no such reduction was observed in the In-field group. This finding suggests the involvement of non-targeted effects of radiation mediated by intercellular communications between the In-field and Out-of-field cells. It appears that signals that decrease the migration velocity are transmitted from the In-field to Out-of-field cells, while signals that increase the velocity are transmitted from the Out-of-field to In-field cells (Figure 5A). Notably, the suppression of the Out-of-field velocity may contribute to the control of tumor invasion and metastasis beyond the planning target volume (PTV) in radiotherapy.

Despite the observed reductions in migration velocity and total distance, the mean squared displacement (MSD) increased linearly over time in all groups, indicating that cells exhibited random walk migration patterns up to 24 h after irradiation. Furthermore, no significant differences were found in net displacement, defined as the straight-line distance between the starting and ending positions of each cell. These results indicate that cell migration remained non-directional, suggesting that intercellular interactions and chemotactic responses to irradiation-induced signals were weak or absent.

With cisplatin treatment, a significant increase in migration velocity was observed in the Uniform, In-field, and Out-of-field cells, compared to the Control after irradiation. The enhanced velocity of the Uniform cells is consistent with the previous study, supporting the reproducibility [5]. Similarly, a significant increase in the total distance traveled was observed in the Uniform, In-field, and Out-of-field cells after irradiation compared to the Control. It appears that signals that increase the migration velocity are transmitted from the In-field to Out-of-field cells, while signaling from the Out-of-field to In-field cells remains unclear (Figure 5B). A noteworthy finding is the increased migration velocity and total distance traveled of the Out-of-field cells, which suggests that radiochemotherapy may promote tumor invasion and metastasis beyond the PTV. However, it remains uncertain whether this enhancement reflects a direct modulation of non-targeted effects by cisplatin itself or instead results from altered cellular responsiveness due to the accumulation of cells in G2 phase induced by cisplatin prior to irradiation. It has been reported that ovarian cancer cells treated with cisplatin release extracellular vesicles, which can induce their invasiveness [14]. Although further investigations are required to elucidate the underlying mechanisms, unraveling and regulating the mechanisms of this enhancement will be a turning point for the advancement of radiochemotherapy.

Similarly, MSD analysis showed a linear increase over time in all groups, consistent with random walk behavior. While net displacement remained unchanged in the Uniform and In-field groups, it was significantly increased in the Out-of-field group compared to the Control. These results suggest that, although cisplatin may have selectively enhanced directionally persistent migration in unirradiated cells located outside the radiation field, overall migration remained largely non-directional. Thus, intercellular interactions and chemotactic responses to irradiation-induced signals appear to be weak or absent, even in the presence of cisplatin.

In previous studies, microbeam irradiation of colonies composed of 20–80 HeLa-FUCCI cells showed that although progeny of bystander cells exhibited cell death or cell cycle arrest, the cell cycle distribution of the bystander cells themselves was not significantly affected [15]. Furthermore, another study using microbeam irradiation reported that intercellular regulation of the cell cycle could not be detected in HeLa-FUCCI cells [16]. As demonstrated by these results, non-targeted effects on cell cycle regulation have not been observed in HeLa-FUCCI cells. Interestingly, however, the findings of the present study suggest the presence of non-targeted effects on cell migration. This discovery may provide clinically important implications for the optimization of radiochemotherapy.

One of the technical limitations is that this study was conducted using only the combination of HeLa-FUCCI cells and cisplatin. HeLa-FUCCI cells were selected as a well-established model system that enables stable visualization of cell cycle progression. However, to evaluate the universality of the observed phenomena, future studies using other cell lines derived from different tissues, in combination with other anticancer agents, will be necessary. The heterogeneous characteristics of tumor cells, such as cancer stem cells, are also important factors in cancer development [17], and validation using mixed cultures with multiple cells may be beneficial. In addition, our evaluation of migration relied solely on single-cell tracking. Future studies should incorporate complementary methods—such as the wound healing assay to evaluate collective migration and the Transwell assay to measure invasive potential—to achieve a more comprehensive understanding. Furthermore, analysis of migration-related markers by Western blotting will be essential to elucidate the underlying molecular mechanisms. Another limitation lies in the use of two-dimensional (2D) cell culture systems, which do not fully capture the complex biological context of clinical environments. In particular, the dynamic signaling crosstalk between cells and the extracellular matrix is only partially reproduced in 2D culture models [18]. Therefore, future studies should incorporate three-dimensional (3D) cell culture systems or organoids, which better mimic the physiological conditions observed in vivo. The quantitative analysis of such advanced models could be facilitated by our framework, as the Cellpose algorithm integrated within TrackMate supports 3D cell detection and tracking [7], making it a potentially powerful tool for future invasion assays. Furthermore, the use of intensity-modulated radiotherapy or spatially fractionated radiotherapy, such as microbeam radiotherapy [19], which allows for more heterogeneous exposure modalities, may help to better define the benefits or risks of non-targeted effects in cancer treatment.

## 4. Materials and Methods

### 4.1. Cell Culture

HeLa-FUCCI cells (RCB2812; RIKEN BioResource Center, Tsukuba, Ibaraki, Japan) [6] were maintained in low-glucose Dulbecco’s modified Eagle’s medium (DMEM; FUJIFILM Wako Pure Chemical, Osaka, Japan), supplemented with 10% fetal bovine serum (Equitech-Bio Inc., Kerrville, TX, USA) and 1% penicillin-streptomycin (Sigma, St. Louis, MO, USA) at 37 °C with 5% CO_2_. The doubling time was approximately 18–20 h, and cells were passaged to maintain a confluence rate of 30–60%.

### 4.2. Drug Treatment

Cells were treated with 5 μM cisplatin (Selleck Chemicals, Houston, TX, USA) in culture medium for 1 h. Following treatment, the medium was replaced with fresh drug-free medium, and the cells were incubated for an additional 14 h prior to irradiation. This treatment led to G2 phase arrest in approximately 70.3% of the cell population.

### 4.3. FUCCI Fluorescence Imaging

An All-in-One BZ-9000 Fluorescence Microscope (Keyence, Osaka, Japan) was used, with excitation and emission wavelengths set to 470 nm and 535 nm for green fluorescence and 540 nm and 605 nm for red fluorescence, respectively.

### 4.4. X-Ray Settings

**Figure 6:**
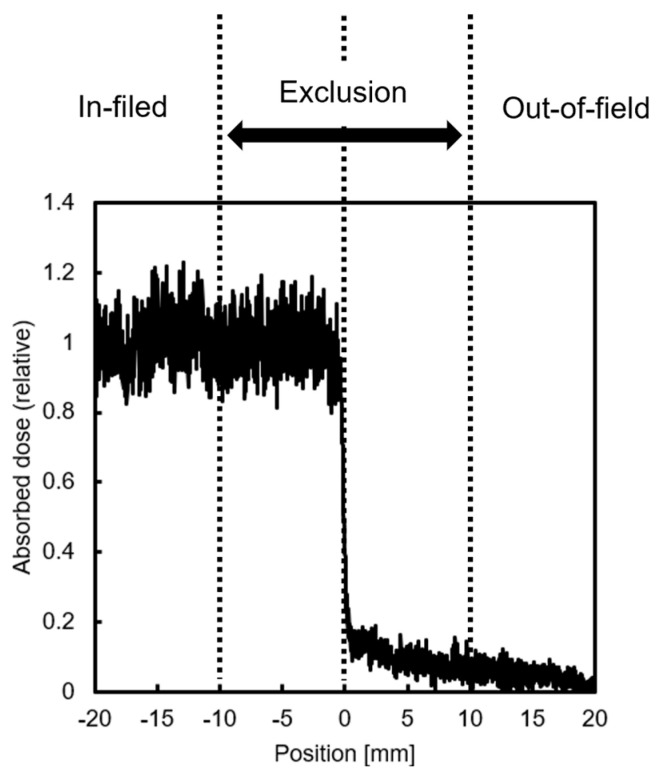
Dose profile of half-field irradiation. Cells within a 20 mm “exclusion region” around the boundary were not included in the analysis.

In this study, half of the dishes seeded with HeLa-FUCCI cells were shielded with Pb blocks and irradiated with 2 Gy of X-rays to divide them into irradiated (In-field) and non-irradiated (Out-of-field) areas. Sham-irradiated (Control) and uniformly irradiated (Uniform) dishes were also prepared for comparison analysis. Gafchromic™ RTQA2 (Lot #: 11032202; Ashland Inc., Wayne, NJ, USA) was used to evaluate the dose near the boundary between In- and Out-of-field areas (Figure 6). Cells within a 20 mm “exclusion region” were not included in the analysis to account for uncertainties in the setup and to avoid analyzing areas with steep dose gradients. Doses delivered via secondary electrons scattering from the exposed region were excluded from consideration. X-ray irradiation was carried out using a 150 kVp beam filtered through 1.0 mm aluminum (Al), delivered by an MBR-1520R-4 generator (Hitachi Power Solutions, Ibaraki, Japan) at a dose rate of 1.83 Gy/min.

### 4.5. Deep Learning-Based Imaging Analysis

A key parameter for characterizing cell migration following irradiation is the two-dimensional (2D) cell velocity (μm/h). To assess this, brightfield images of irradiated cells were captured at 15 min intervals over a 24 h period using a time-lapse microscope (WSL-1800, ATTO, Tokyo, Japan) with an exposure time of 10 ms. The acquired images were subsequently processed and compiled into time-lapse sequences for analysis.

Cell segmentation in this study was performed using the Cellpose framework, specifically leveraging the pre-trained Cytoplasm 2.0 model. This model was further refined through fine-tuning with a training dataset comprising 10 images, each containing 100–200 manually segmented cells. The acquisition conditions for these training images were maintained consistent with those used for time-lapse imaging. Subsequent analysis of the time-lapse sequences was conducted using ImageJ 1.54f/Fiji (Java 1.8.0_322) [20]. This version of Fiji incorporates TrackMate 7.11.1 [11], an automated image analysis tool for biological applications, which seamlessly integrates Cellpose 2.0 [8], an advanced algorithm optimized for segmenting cells in brightfield microscopy images.

The mean square displacement (MSD) is one of indicators of cell movement mechanisms. Given a stochastic process $x_{i}\left(t\right)$, the $MSD\left(t\right)$ is calculated as follows [21]:

(1) $$MSD\left(t\right)=\frac{1}{N}\sum_{i=1}^{N}{\left[x_{i}\left(t\right)-x_{i}\left(0\right)\right]}^{2}$$

where N is the total number of particles, $x_{i}\left(t\right)$ is the position of particle i at time t, and $x_{i}\left(0\right)$ is the position of particle i at the initial time.

In any dimension ≥ 2, the MSD of a random walk in a heterogeneous environment exhibits a linear dependence on time at sufficiently long timescales [22]. In this study, the MSD was computed based on the displacement of cells at each time point, and the extent to which cell movement conformed to a random walk was inferred from the linearity of the MSD. The degree of linearity was quantified using the coefficient of determination ($R^{2}$) obtained from the data and linear fitting.

### 4.6. Statistical Analysis

For each experimental group, over 30 cells were analyzed across a minimum of three independent replicates. Cells that exited the observation field or appeared nonviable during imaging were excluded from analysis due to incomplete trajectory data. Migration velocity was evaluated over a 24 h period. Statistical comparisons between the Control and other groups were performed using Steel’s test. In addition, a chi-square test was used to assess differences in the distribution of cell cycle phases based on FUCCI fluorescence. A *p*-value below 0.05 was considered indicative of statistical significance. All analyses were conducted using Python Anaconda version 23.3.1.

## 5. Conclusions

Using cell segmentation and tracking algorithms, Cellpose 2.0 and TrackMate 7, deep learning-based imaging analysis showed the non-targeted effects of radiation on cancer cell migration and the modification of cisplatin. The increased migration velocity of the Out-of-field cells with cisplatin treatment indicated that chemoradiotherapy can promote tumor invasion and metastasis beyond the PTV. These findings may aid in refining treatment protocols that integrate radiation therapy with anticancer agents, potentially enhancing therapeutic efficacy.

## Data Availability

All data presented in this study are contained within the article. For any further information, please contact the corresponding author.
